# Embryonic stem cells overexpressing high molecular weight FGF2 isoform enhance recovery of pre-ganglionic spinal root lesion in combination with fibrin biopolymer mediated root repair

**DOI:** 10.1186/s13287-024-03676-6

**Published:** 2024-03-05

**Authors:** B. H. M. Lima, L. P. Cartarozzi, S. Kyrylenko, R. S. Ferreira, B. Barraviera, Alexandre L. R. Oliveira

**Affiliations:** 1 Department of Structural and Functional Biology, Laboratory of Nerve Regeneration, Institute of Biology, University of Campinas, Campinas, 13083-862 SP Brazil; 2 Biomedical Research Center, Medical Institute of Sumy State University, Sumy, 40018 Ukraine; 3 Center for the Study of Venoms and Venomous Animals (CEVAP), São Paulo State University (UNESP), Botucatu, 18610-307 SP Brazil

**Keywords:** Cell therapy, Embryonic stem cells, Fibroblast growth factor 2, Motoneurons, Spinal cord injury

## Abstract

**Background:**

Spinal ventral root avulsion results in massive motoneuron degeneration with poor prognosis and high costs. In this study, we compared different isoforms of basic fibroblast growth factor 2 (FGF2), overexpressed in stably transfected Human embryonic stem cells (hESCs), following motor root avulsion and repair with a heterologous fibrin biopolymer (HFB).

**Methods:**

In the present work, hESCs bioengineered to overexpress 18, 23, and 31 kD isoforms of FGF2, were used in combination with reimplantation of the avulsed roots using HFB. Statistical analysis was conducted using GraphPad Prism software with one-way or two-way ANOVA, followed by Tukey’s or Dunnett’s multiple comparison tests. Significance was set at *p < 0.05, **p < 0.01, ***p < 0.001, and ****p < 0.0001.

**Results:**

For the first set of experiments, rats underwent avulsion of the ventral roots with local administration of HFB and engraftment of hESCs expressing the above-mentioned FGF2 isoforms. Analysis of motoneuron survival, glial reaction, and synaptic coverage, two weeks after the lesion, indicated that therapy with hESCs overexpressing 31 kD FGF2 was the most effective. Consequently, the second set of experiments was performed with that isoform, so that ventral root avulsion was followed by direct spinal cord reimplantation. Motoneuron survival, glial reaction, synaptic coverage, and gene expression were analyzed 2 weeks post-lesion; while the functional recovery was evaluated by the walking track test and von Frey test for 12 weeks. We showed that engraftment of hESCs led to significant neuroprotection, coupled with immunomodulation, attenuation of astrogliosis, and preservation of inputs to the rescued motoneurons. Behaviorally, the 31 kD FGF2 - hESC therapy enhanced both motor and sensory recovery.

**Conclusion:**

Transgenic hESCs were an effective delivery platform for neurotrophic factors, rescuing axotomized motoneurons and modulating glial response after proximal spinal cord root injury, while the 31 kD isoform of FGF2 showed superior regenerative properties over other isoforms in addition to the significant functional recovery.

**Supplementary Information:**

The online version contains supplementary material available at 10.1186/s13287-024-03676-6.

## Introduction

Preganglionic nerve root injuries have been considered untreatable for the most part due to the challenging surgical approach necessary to reattach the ruptured rootlets to the surface of the spinal cord, together with the long distance that regrowing axons need to travel toward the target. Nerve root injuries have been documented since the 19th century, predominantly affecting the brachial plexus [1], including high-energy traumatic events such as automobile accidents, particularly involving motorcycles [2]; and iatrogenic factors associated with surgical procedures [3]. These injuries often result in significant and permanent loss of function, together with muscle atrophy and severe pain. Overall, such outcome leads to poor quality of life, depression, and elevated treatment costs [4, 5].

The first step towards developing efficient treatment approaches to plexus injuries is the establishment of animal models. In this regard, models of neonatal peripheral nerve injuries have been considered, since during the first week of postnatal life, axotomy at the mid-tight level results in loss of up to 70% of the motoneurons in the spinal cord [6–10]. However, these models face important limitations regarding regeneration and glial reaction due to the immaturity of the nervous system. Thus, the use of adult rats has been proposed [11] together with surgical reconstruction of the ruptured roots [12–16].

Although pial suture was proposed to reattach avulsed roots to the surface of the spinal cord, the use of a biological glue, the heterologous fibrin biopolymer (HFB), has been considered efficient, allowing the reimplantation of multiple rootlets [15, 17]. In fact, a recent review by Buchaim et al., (2019) [18] on HFB, emphasized its promising hemostatic, adhesive, drug delivery, and scaffold (support structure) properties. The scaffold property, in particular, facilitates advanced therapies including stem cells (SC).

Cell therapy represents a recent and promising approach that can be combined with surgery to repair the CNS/PNS interface [19]. Ribeiro et al. (2015) [20] investigated the neuroprotective effects of xenotransplantation of mesenchymal stem cells derived from adipose tissue in rats following ventral root avulsion, which led to increased neuronal survival. Moreover, human mesenchymal stem cells (hMSCs) derived from adipose tissue, bone marrow, and dental pulp have been observed to possess immunomodulatory effects [21–23]. In these studies, SCs were found to influence the relative expression of pro-and anti-inflammatory cytokines and neurotrophic factors.

Embryonic stem cells (ESCs) have also been investigated for their therapeutic potential [24–26]. Unlike mesenchymal stem cells, ESCs are characterized as pluripotent, which in turn may provide further tools for regeneration [27]. Additionally, recent cell culture techniques enabled long-lasting expansion of these cells without chromosomal, mitochondrial or epigenetic changes [28, 29] and without using xenoderivatives (such as the murine embryonic fibroblast monolayer or MEF) that pose a risk of pathogen transmission [30]. Moreover, by utilizing genetic manipulation techniques, human embryonic stem cells (hESCs) can be bioengineered to overexpress trophic factors such as basic fibroblast growth factor 2 (FGF2) [31].

Basic fibroblast growth factor 2 (FGF2) is a potent mitogen essential for the survival of many cell types [29]. It is believed that the translation of human FGF2 gene results in several major protein isoforms with variations in molecular weight. While the low molecular weight (LMW) isoform (18 kD) is mostly cytoplasmic, the high molecular weight (HMW) counterparts with apparent molecular weights of 22, 22.5, 24, and 34 kD are translocated to the nucleus [32]. According to UNIPROT data (uniprot.org/uniprotkb/P09038) the molecular weights of the isoforms of the human FGF2 are 17,254 kD (isoform 3, 155 aa); 21,203 kD (isoform 4, 196 aa); 22,623 kD (isoform 2, 210 aa); and 30,770 kD (isoform 1, 288 aa). In this work, we used the sequences of the 3 human FGF2 isoforms of 17,254 kD, 22,623 kD, and 30,770 kD and refer them to as isoforms 18 kD, 23 kD, and 31 kD, respectively.

Both LMW and HMW isoforms of FGF2 are considered therapeutic modalities in neurodegeneration models [33]. The HMW isoform of FGF2 is involved in cellular responses to injury and is upregulated in several pathologies such as cancer and cardiovascular diseases [34]. The regulation of the HMW FGF2 isoform is not fully understood, but it is known to be under the control of a complex system involving various transcription factors and signaling pathways. Different isoforms of FGF2 can be regulated by alternative promoter usage, alternative splicing, and alternative polyadenylation. Additionally, FGF2 expression can be regulated by epigenetic factors such as DNA methylation and histone modification. Understanding the regulation of the HMW FGF2 isoform gene could have important implications for the treatment of several diseases and for the development of new therapeutics.

In this study we compared different isoforms of FGF2 (18, 23, and 31 kD), inducibly overexpressed in stably transfected hESCs, following motor root avulsion and repair with HFB. The initial goal was to evaluate which isoform would provide greater neuroprotection, in order to determine which hESC would be more effective in rescuing and stimulating regrowth of motor axons after axotomy. We showed that the isoform of FGF2 with the highest molecular weight (31 kD) provided the best morphological and functional results. This could offer a platform for further investigation towards spinal cord repair after injury, as well as prospects for future translation to the clinic.

## Materials and methods

### Experimental design

Adult female Lewis rats, nine weeks old on average and weight around 200 g, were obtained from the Multidisciplinary Center for Biological Investigation (CEMIB, Brazil) at the University of Campinas. The experimentation was approved by the Animal Experimentation Ethics Committee of the Institute of Biology at the State University of São Paulo (CEUA/IB/UNICAMP, protocol number 5225-1/2019), and conducted following the guidelines of the National Council for the Control of Animal Experimentation (CONCEA). All procedures involving animals were designed to minimize pain and discomfort and were carried out under the supervision of qualified veterinarians. The study adheres to the principles of the 3Rs (Replacement, Reduction, and Refinement) to ensure ethical treatment of the animals used.

The animals were kept in standard polysulfone cages (dimensions: 240 mm x 251 mm x 386 mm), with a maximum of two animals per cage to allow adequate space and social interaction. The cages were equipped with environmental enrichment, to promote natural behaviors and well-being. The housing room was maintained at a temperature of 22 ± 2 °C with a relative humidity of 50 ± 10%. A 12-hour light-dark cycle was established to mimic natural circadian rhythms, with free access to water and pelleted food.

The experiments were divided into two sets. In the first set, we used three cell lines of hESCs stably overexpressing different FGF2 isoforms (18, 23, and 31 kD) and identified which cell line had the best neuroprotective effect and gliosis modulation after motor root avulsion without reimplantation. In the second set of experiments, once the most effective isoform of the FGF2 was identified (31 kD), new groups underwent avulsion, but this time with reimplantation using HFB. A more detailed analysis was performed through immunohistochemistry, qRT-PCR, and functional analysis (CatWalk walking track and von Frey tests).

A total of 95 animals were used and distributed according to Table 1. Such sample size was determined based on power analysis calculations to ensure sufficient statistical power for detecting significant differences between experimental groups. Additionally, the sample size was chosen to balance the need for robust and reliable data with the ethical consideration of using the minimum number of animals necessary to achieve meaningful scientific outcomes.

Additionally, this study adheres to the ARRIVE guidelines for the reporting of animal experiments, ensuring the transparent and comprehensive reporting of the methods as well as handling of the subjects. The funding agencies played no role in the design of the study and collection, analysis, and interpretation of data and in writing the manuscript.

**Table 1:** Experimental design

	Applied techniques	Motorneuron survival		Immuno- histochemistry	Synaptic mapping		qRT-PCR	Functional recovery	
								Catwalk	Von Frey test
	Experimental groups/ Time	2 weeks	12 weeks	2 weeks				12 weeks	
1st Experiment	Av + HFB + Dox	5^a^	-	5^a^		-	-	-	-
	Av + HFB + Dox + hESC **Wild Type**	5^a^	-	5^a^		-	-	-	-
	Av + HFB + Dox + hESC **18 kD**	5^a^	-	5^a^		-	-	-	-
	Av + HFB + Dox + hESC **23 kD**	5^a^	-	5^a^		-	-	-	-
	Av + HFB + Dox + hESC **31 kD**	5^a^	-	5^a^		-	-	-	-
2nd Experiment	AvR + HFB + Dox	-	5^c^	5^b^		5^b^	5	5^c^	5^c^
	AvR + HFB + Dox + **Commercial FGF2**	-	5^c^	5^b^		5^b^	5	5^c^	5^c^
	AvR + HFB + Dox + **hMSC**	-	5^c^	5^b^		5^b^	5	5^c^	5^c^
	AvR + HFB + hESC 31kD **OFF**	-	5^c^	5^b^		5^b^	5	5^c^	5^c^
	AvR + HFB + **Dox** + hESC 31kD	-	5^c^	5^b^		5^b^	5	5^c^	5^c^

The study was divided into two sets of experiments: the first one focused on neuronal survival in rats subjected to ventral root avulsion, without reimplantation, and was dedicated to establishing which isoform of FGF2 was the most effective in rescuing motor neurons following an axonal injury. The second set of experiments was dedicated to the detailed evaluation of the most neuroprotective isoform of FGF2 (31kD). Assessment of the neuronal microenvironment and functional recovery was conducted, with comparisons made to control groups. The groups assembled for evaluation in both experiments are listed. a: the same rats were used for both motoneuron survival and immunohistochemistry; b: the same rats were used for both immunohistochemistry and synaptic mapping; c: the same rats were used for both 12 weeks motoneuron survival and functional recovery. Av: Avulsion; AvR: Avulsion and reimplantation; Dox: Doxycycline used to induce FGF2 overexpressing genetic construct.; HFB: Heterologous fibrin biopolymer; hESC: Human embryonic stem cell; hMSC: Human mesenchymal stem cell

### Ventral roots avulsion

Anesthesia was induced with ketamine (100 mg/Kg) and xylazine (10 mg/Kg) and the rats were maintained in a surgical plane with 2% isoflurane. Bepanthen (Bayer, Germany) was applied to the eyes to prevent dryness. The animals were shaved on their backs and gently placed on a heated pad (36 °C) in the prone position. A surgical incision was made in the craniocaudal axis at the thoracolumbar level, parallel to the spine, allowing the retraction of the paraspinal muscles and exposure of the vertebrae. Laminectomy was performed on approximately three vertebrae, and the dura mater was accessed with a longitudinal incision, allowing exposure to the lumbar enlargement. The denticulate ligament was dissected, enabling the manipulation and location of the ventral roots in the intervertebral region. Unilateral avulsion of the L4, L5, and L6 roots on the right side was performed by gently applying traction with fine forceps #4 (Dumont®, Switzerland, Part number 11242-40). After the surgical procedures, the roots were repositioned, and the musculature, fascia, and skin were sutured in layers. Tramadol hydrochloride (Germed Farmacêutica Ltda, Hortolândia/SP, Brazil) was administered orally by gavage at a concentration of 5 mg/kg every 12 h for 72 h following surgery.

### Root replantation and HFB

In the first set of experiments, the avulsed ventral roots were positioned on the muscle bed in such a way that they could not rejoin the spinal cord, followed by the application of HFB in the same region. In contrast, for the second set of experiments, the avulsed roots were gently repositioned as close as possible to their original location on the surface of the lumbar intumescence, followed by the application of HFB for coaptation. HFB is composed of three fractions that are homogenized and applied in sequence, at the lesion site in a total volume of 6 µL: (I) fibrinogen cryoprecipitate derived from the blood of *Bubalus bubalis* (3 µL), (II) calcium chloride diluent (2 µL), and (III) gyroxin, a thrombin-like enzyme from the *Crotalus durissus terrificus* snake (1 µL) [35–37]. The third fraction added is responsible for the polymerization process, allowing the coaptation and stabilization of the roots, which can be observed during the surgical process [38–40]. HFB components and application formulas are listed in its patent (BR1020140114327) and were kindly provided by the Center for the Study of Venoms and Venomous Animals (CEVAP/UNESP, Brazil).

### Bioengineered embryonic stem cells

The hESCs used in this study belong to the CCTL12 lineage, originating from Masaryk University in Brno, Czech Republic. The cells were modified into three different cell lines, each stably overexpressing a different isoform of human FGF2: 18 kD, 23 kD, and 31 kD in a doxycycline-inducible manner. Cells of each subtype were cultured separately (as described below) and applied to the lesion site in conjunction with components of HFB, before adding gyroxin responsible for initiating the polymerization process of the HFB.

### Establishment of the genetic vectors and transgenic hESCs

Araújo et al., (2017) [31] provided a detailed description of the establishment of the transgenic cells used herein. Briefly, to amplify DNA fragments for cloning and/or transfection, Phusion High Fidelity DNA Polymerase (Finnzymes/Thermo Fisher Scientific, Vantaa, Finland) was utilized. In order to obtain stable hESC lines that overexpress GFP-fused various isoforms of FGF2 in an inducible manner, the Tet-On system (Clontech/Takara Bio, Mountain View, CA, USA) was employed. Vectors for 23 kD and 31 kD isoforms of FGF2 were constructed similarly as described in Araújo et al., (2017) for the isoform 18 kD. For the highest molecular weight isoform 31 kD, a fragment of the 5’ part of the ORF was chemically synthesized (DNA2.0, Menlo Park, CA, USA) with optimized codon usage to reduce GC content because the 5’ part of the wild type human FGF2 gene has GC content too high to be amplified by PCR. The full sequences of the resulting vectors pTe106, pTe105, and pTe95 for 18 kD, 22.6 kD, and 31 kD isoforms, respectively, are accessible from the GenBank (accession numbers KX844812, OQ982570, and OQ982569).

The vectors obtained by preparative PCR were used for stable cell transfections. Cell selection was performed using the antibiotics G-418 at a concentration of 140 µg/mL and blasticidin S at a concentration of 1.2 µg/mL. Cells were transfected, seeded, and selection was conducted for two weeks with regular medium changes. Induction of transgene expression was achieved by treating the cells with 1 µg/mL of doxycycline (Dox) for 48 h. The resulting stable cell clones, named E12-1-1 (18kD), E12-2-1 (23kD), and E12-3-1 (31kD), which inducibly express each of the fused forms of human FGF2 with GFP, underwent double selection and were used in further experiments. Cell karyotypes were confirmed by the Institut für Humangenetik und Anthropologie, Jena, Germany.

### Obtaining the hMSC

The adipose tissue-derived hMSCs were obtained through liposuction, with prior consent from the donor and approval for the use of the cells (CAAE: 1162.0.146.000–11). The steps involved in cell collection, extraction, and cultivation were performed at the Cellular and Molecular Biology Laboratory, located at the Blood Center - UNICAMP [20, 21, 41], under the supervision of Dr. Ângela Cristina Malheiros Luzo, the medical director of the Umbilical Cord and Human Placenta Blood Bank and Transfusion Service.

### Cell culture

Bioengineered hESCs were cultured in monolayers on well plates coated with Matrigel (Corning Life Sciences, USA, code 354,277), following the method described by Kunova et al., (2013) [42]. Cultivation was carried out in mTeSR™1 medium (Stemcell Technologies™; code 85,850) at a temperature of 37 °C and in an atmosphere with 5% CO_2_ until the cells were semi-confluent. To induce the overexpression of FGF2 in the cells, Dox (Doxycycline hyclate, Sigma) was added to the culture medium at a final concentration of 1 µg/mL, 24 h before harvesting. The cells were then detached with ReLeSR (StemCell Technologies, enzyme-free hESC and hiPSC selection and passaging reagent; code #05872), washed, and counted in a Neubauer chamber.

The hMSCs were kept under conditions of temperature, humidity, and CO_2_ identical to those used for the hESCs. However, they were cultured on uncoated plates and expanded in Dulbecco’s Modified Eagle Medium (DMEM) containing 10% fetal bovine serum and 1% penicillin and streptomycin until they reached approximately 80% confluence.

### Transplantation of hESCs and hMSCs into the HFB scaffold

Immediately after ventral root avulsion and before the administration of gyroxin (HFB polymerization component), approximately 3 × 10^5^ hESCs or hMSCs were resuspended in 3–5 µl of mTeSR™1 or DMEM, respectively, and transplanted directly onto the lesion site. As previously described, to induce overexpression of FGF2 in vitro in hESCs, doxycycline was added to the culture medium. The effectiveness of the induction was confirmed by the expression of GFP (green) as shown in Supplementary Fig. 1. For in vivo experiments, Dox was administered to the animals along with food at a concentration of 625 mg of Dox per kg of pelletized feed. In animals to which commercial FGF2 (recombinant human FGF-basic - rhFGF2, BioLegend catalog no. 710,304) was applied, rhFGF2 was administered directly, along with HFB, similarly to the cell therapy counterparts. For that, 3 µl of rhFGF2 (10 µg/µl) was used [43].

### Euthanasia and specimen preparation

Rats were perfused according to survival time points for analysis. For this purpose, they were subjected to an overdose of xylazine and ketamine and underwent thoracotomy followed by transcardiac perfusion with a 0.1 M sodium phosphate-buffered saline solution (PBS) containing 0.9% sodium chloride (NaCl) (pH 7.38). Subsequently, samples designated for motoneuron survival, immunohistochemistry, and behavioral analysis were perfused with a 4% paraformaldehyde-fixative solution in PBS 0.1 M. After fixation, the lumbar enlargement was carefully dissected out and immersed in the same fixative solution overnight at 4 °C, washed three times with 0.1 M PBS and immersed in sucrose solutions (10%, 20%, and 30%, each for 12 h). Finally, they were covered with Tissue-Tek and frozen in n-hexane under controlled temperature (-32 °C to -35 °C) and stored at -20 °C. In contrast, samples for qRT-PCR were not fixed and remained stored in an ultrafreezer at -80 °C immediately after perfusion with 0.1 M PBS. The complete workflow is detailed in Supplementary Fig. 1.

### Motoneuron survival

The cryopreserved specimens (2 and 12 weeks after injury) were submitted to cryostat (Micron, HM525) sectioning (12 μm thick). Motoneuron counts were conducted in cross-sections of the lumbar enlargement, stained with Nissl (0.05% toluidine blue in distilled water for 1 min, rinsed, dehydrated in alcohol, and coverslip mounted with Entellan (Merck). Motoneurons located in the lateral motor nucleus of the ventral horn, lamina IX of Rexed, were counted on the ipsilateral (injured) and contralateral (uninjured) sides, with an interval between sections of approximately 480 μm. Only cells with visible nucleus and nucleolus were considered for counting.

To avoid double counting of neurons, resulting from the fact that the same cell is present in two sections, the formula described by Abercrombie; Johnson, (1946) [44] was used. The mean ± standard error of the mean (SEM) ratio of data collected per animal (ipsilateral/contralateral side) was calculated for each group.

### Immunohistochemistry

Cross-sections of the lumbar enlargement, 12 μm thick, were obtained using a cryostat (Microm, HM525), transferred to gelatin-coated glass slides, and stored at -20 °C until analysis. To perform immunohistochemistry, the slides were brought to room temperature, and the sections were delineated with a hydrophobic pen (PAP pen, Sigma Z377821). Subsequently, the slides were placed in a humidity-controlled chamber, protected from light. Sections were immersed in 0.01 M PBS (3 × 5 min each, pH 7.38), and incubated in 150 µL of blocking solution (3% bovine serum albumin in 0.1 M PBS, pH 7.38) for 45 min. Following this step, the primary antibodies were diluted in an incubation solution (1.5% bovine serum albumin and 0.2% Tween in 0.1 M PBS, pH 7.38) overnight at 4 °C.

After incubation with the primary antibody, sections were washed with 0.01 M PBS and incubated at room temperature with the appropriate secondary antibody for 45 min. The sections were then washed with 0.01 M PBS, dried, and cover-slipped using a glycerin/PBS (3:1) mounting medium. The slides were examined using an epifluorescence microscope (Leica DMB5500) and documented with a digital camera (Leica DFC 345 FX) equipped with specific filters, depending on the secondary antibodies used. The antibodies used are detailed in the Supplementary Table 1.

### Integrated density of pixels quantification

The integrated density o pixels, which indicates the intensity of immunostaining, was measured in the lateral motor nucleus of the ventral horn ipsilateral and contralateral to the lesion in the images obtained by immunohistochemistry [45, 46]. The evaluation of the integrated density of pixels was performed in three representative images obtained from each animal, at different levels of the lumbar enlargement (L4-L6) using ImageJ software (version 1.33 u, National Institutes of Health, Bethesda, MD, USA). In summary, the 8-bit images were subjected to threshold segmentation, with the cutoff value defined by comparing the image with its RGB counterpart. For the quantification of Iba-1, GFAP, VGLUT1, and GAD65, the total area of the 20x image was used; for anti-synaptophysin immunostaining, eight equidistant and predefined circular areas around each motoneuron (at 40x) were measured, according to Freria et al., (2012) [47]. The mean ± SEM data collected per animal (ipsilateral/contralateral side) was calculated for each group, except for GAD65, in which the values of each spinal cord side were presented separately.

### Analysis of gene expression by qRT-PCR

Total RNA extraction was performed using the Qiazol Lysis Reagent (Qiagen - cat. no. 79,306), followed by the synthesis of complementary DNA (cDNA) using the High-Capacity cDNA Reverse Transcription Kit (Applied Biosystems, catalog number: 4,368,814), with 2.0 µg of total RNA according to the manufacturer’s instructions. Subsequently, the real-time PCR reaction was carried out using the TaqMan assay (Life Technologies) to assess the relative levels of gene expression of the genes described in Table 2.

**Table 2:** qRT-PCR analysis

Gene	Code		Gene
GAPDH	Rn01775763_g1	Rn02531967_s1	BDNF
HPRT1	Rn01527840_m1	Rn00569510_m1	GDNF
IL-1β	Rn0058042_m1	Rn00566673_m1	HGF
IL-6	Rn01410330_m1	Rn00570809_m1	FGF2
TNFα	Rn01525859_g1	Rn01511602_m1	VEGFa
TGFβ	Rn00572010_m1	Rn00560865_m1	B2m
IL-10	Rn99999012_m1	Rn00596773_m1	CD3ζ (CD247)

TaqMan assays used and their respective codes

Reactions were performed in triplicate, using cDNAs, TaqMan® Gene Expression Master Mix (2x) (Life Technologies - PN 4,369,016), RNase-free water, and Taqman assays containing primers and probes for the mentioned genes. Thermal profile was set to: 95 °C for 10 min + 45 cicles of 95 °C for 15 s and 60 °C for 1 min. The HPRT1 reference gene was selected because it exhibited unaltered expression under various experimental conditions. FAM and VIC fluorophores were used to identify the genes of interest and the reference gene, respectively. The complete quantitative PCR protocol was carried out with the MX3005P instrumentation platform (Agilent, Santa Clara, CA, USA), and the results were analyzed using MxPro software (Agilent). For statistical analysis, the mean values of the three measurements performed for each animal were used as individual data for the relative quantification of the genes of interest, employing the 2^−ΔΔCt^ method [48].

### Functional gait and nociceptive recovery

Gait-related data acquisition was performed using an automated gait walkway system (CatWalk System, Noldus Inc., the Netherlands), where animals moved freely without reward stimuli. For each animal, three runs were recorded using a high-speed capture camera (Fujinon DF6H-1B) equipped with a wide-angle lens (8.5 mm, Fujicon Corp., China). Data were stored and analyzed using CatWalk XT 10.5 software (Noldus Inc.). The animals were acclimatized, and their runs were recorded before the injury (baseline) and starting from the third day after the root avulsion. Gait functional analysis data were collected weekly for 12 weeks (84 days). The parameters were set as follows: 0.50 to 5.00 s of run duration, 60% speed variation, 25.01 camera gain, and 0.25 detection limit.

Gait quality was calculated using the regression analysis proposed by the peroneal functional index (PFI) established by Bain; Mackinnon; Hunter, (1989) [49], which takes into account the length of the paw, the distance between the first and fifth toes on the injured and non-injured side, in addition to other measures such as max contact area (cm²), stand (s), max intensity at (%), swing (s), step cycle (s), and base of support (cm).

An electronic esthesiometer, originally developed for use in humans [50] and later adapted for evaluation in rats [51] was used to evaluate the mechanical nociceptive threshold (von Frey test). The mechanoceptive spinal reflex was evaluated before the injury and once a week until the twelfth week. Pressure was applied to the plantar region, with the tip of a pipette coupled to a force transducer connected to a digital potentiometer that calculated the applied pressure in grams, of the injured right hind paw and to the uninjured contralateral side, with a maximum limit of 90 g to avoid tissue damage. If the animal did not respond to this intensity of pressure, paw anesthesia was considered. The stimulus was repeated until three consistent responses were generated per animal. The paw withdrawal reflex threshold was calculated as the mean ± standard error of the mean ratio of data collected per week (ipsilateral/contralateral side).

### Statistical analysis

All results are presented as mean ± SEM. Statistical analysis of the data was performed using GraphPad Prism software (version 8.0.1 for Windows, GraphPad Software, La Jolla, California, USA). For statistical analysis of motoneuron survival, immunohistochemistry and qRT-PCR, one-way analysis of variance (ANOVA) was used, followed by Tukey’s or Dunnett’s multiple comparison tests. For behavioral tests, two-way ANOVA followed by Dunnett’s multiple comparison test was used. Values of *p < 0.05, **p < 0.01, ***p < 0.001, and ****p < 0.0001 were considered statistically significant.

## Results

### 1st experiment: defining the most effective FGF2 isoform-expressing- transgenic hESCs

The motoneuron survival rate following ventral root avulsion was evaluated two weeks after injury. The groups treated with hESCs expressing the heavier isoforms (Av + HFB + Dox + hESC 23kD and Av + HFB + Dox + hESC 31kD) showed a more pronounced neuroprotective effect (58% ± 3% and 73% ± 4%, respectively) compared to the control group (41% survival ratio), (Av + HFB + Dox x Av + HFB + Dox + hESC 23 kD and Av + HFB + Dox + hESC 31 kD, *p < 0.04 and ****p < 0.0001).

The results indicate that the group treated with wild-type stem cells (AV + HFB + Dox + hESC Wild Type) preserved only 41% ± 4% of motoneurons, which was statistically lower than the groups treated with the heavier FGF2 isoforms (AV + HFB + Dox + hESC Wild Type x Av + HFB + Dox + hESC 23kD and Av + HFB + Dox + hESC 31 kD, with *p = 0.04 and ****p < 0.0001). Furthermore, the 31kD isoform was superior to the 18 kD conunterpart (Av + HFB + Dox + hESC 18kD x Av + HFB + Dox + hESC 31kD, with **p = 0.002).

Synaptic coverage was also affected, with hESC-treated animals expressing the heavy isoform, 31 kD, showing the greatest preservation of synaptic coverage (70% ± 6%), followed by treatment with the 23 and 18 kD isoforms (62% ± 3% and 61 ± 5%). The results were statistically significant when compared to the control group that received only the avulsion (Av + HFB + Dox + hESC 31 kD, Av + HFB + Dox + hESC 23 kD, and Av + HFB + Dox + hESC 18 kD x Av + HFB + Dox; ****p < 0.0001; ***p = 0.0008 and **p = 0.001, respectively). There was no statistical difference in the group treated with non-bioengineered cells as compared to the avulsion-alone counterpart (Av + HFB + Dox + hESC Wild Type).

The glial reaction was upregulated following the lesion so astroglial reactivity increased by 312% as compared with the avulsion-only group (Av + HFB + Dox). When compared with light FGF2 isoform therapy (228% ± 24%), a statistically significant difference was observed (Av + HFB + Dox x Av + HFB + hESC FGF2 18 kD, *p = 0.04). The astroglial reactivity was even more attenuated when heavier FGF2 isoforms were used (Av + HFB + hESC 23 kD and Av + HFB + hESC 31 kD, 204% ± 19% and 206% ± 14%, respectively; **p < 0.01).

**Fig. 1:**
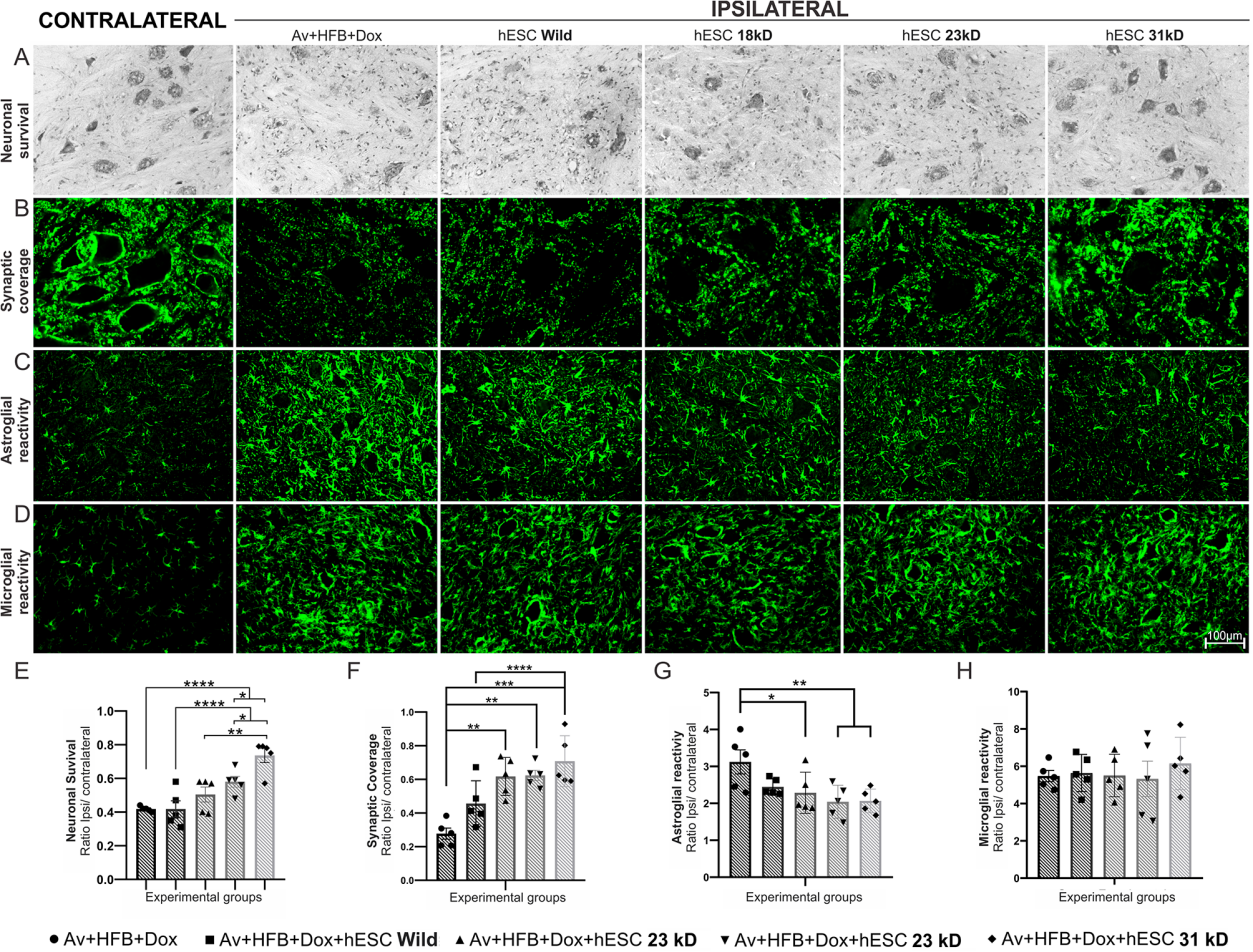
Comparison of hESC isoforms on neuronal and glial outcomes after avulsion without reimplantation. (A – D) Representative images of the spinal cord depicting the contralateral and ipsilateral sides of the injury in the different groups. (E) Enhanced neuronal survival observed predominantly in the group treated with 31 kD hESCs. (F) Preservation of synaptic coverage primarily in the group treated with 31 kD hESCs. (G) Notable astrogliosis control in groups treated with both 23 kD and 31 kD hESCs. (H) The 31 kD hESCs demonstrate a tendency to stimulate microglia, though no significant difference is observed when compared to other groups. Neuronal survival quantification was achieved by counting motor neurons, while synaptic coverage and astroglial and microglial reactivity were assessed using integrated pixel density (ipsilateral/contralateral ratio, *n* = 5 per group). Data are presented as mean ± SEM; * *p* < 0.05; ** *p* < 0.01; *** *p* < 0.001; **** *p* < 0.0001

Microglial reactivity, on the contrary, showed no statistically significant differences among groups, although all of them showed microgliosis after injury (average of 5.6x). The average values and standard error of the mean for Iba-1 staining are summarized in Table 3; Fig. 1.

**Table 3:** Groups and data analyzed in the first set of experiments – avulsion without reimplantation

Groups	Neuronal survival		Synaptic coverage		Astroglial reactivity		Microglial reactivity	
	Mean	SEM	Mean	SEM	Mean	SEM	Mean	SEM
AV + HFB + Dox	0.42	0.01	0.28	0.03	3.12	0.33	5.47	0.30
Av + HFB + Dox + **hESC Wild Type**	0.42	0.05	0.46	0.06	2.45	0.10	5.64	0.44
Av + HFB + Dox + **hESC 18 kD**	0.50	0.04	0.62	0.05	2.29	0.25	5.50	0.51
Av + HFB + Dox + **hESC 23 kD**	0.58	0.03	0.62	0.03	2.04	0.20	5.32	0.95
Av + HFB + Dox + **hESC 31 kD**	0.74	0.04	0.71	0.07	2.07	0.14	6.15	0.63

Neuronal survival, synaptic coverage, astroglial and microglial reactivity 2 weeks following root avulsion in the different experimental groups. Mean ± SEM. Av: Avulsion; HFB: Heterologous fibrin biopolymer; Dox: Doxycycline

### 2nd experiment: high molecular weight isoform of FGF2 is localized predominantly in nucleoli

To shed light on the possible intracellular role of the FGF2 isoforms we investigated their cellular localization. The hESC cells were transiently transfected with the genetic constructs for overexpression of 18 kD, 23 kD, and 31 kD isoforms of FGF2 fused to GFP and were investigated under confocal microscopy after staining the nuclei with DAPI (Supplementary Fig. 2A). The mean fluorescence intensity of the GFP between the nucleoli vs. the rest of the nuclei was determined via the Image J software (Supplementary Fig. 2B). We observed that the HMW FGF2 of 31 kD is localized predominantly in nucleoli, while the lower molecular weight isoforms are distributed all over the nuclei. We suggest that the HMW isoform of FGF2 plays an active role in regulating gene transcription in hESC in comparison to lower molecular weight isoforms.

Additional to the characterization of FGF2 location in the hESCs, we investigated the expression of FGF receptors in the spinal cord ventral horn after avulsion and root repair and observed upregulation of immunolabeling ipsilateral to the injury indicating that FGF2 neurotrophic support is of relevance in such proximal injury scenario (Supplementary Fig. 2C).

### Neuroprotection and glial reaction modulation by hESCs overexpressing 31 kD FGF2

Motoneuron survival was evaluated twelve weeks after ventral root avulsion and reimplantation to access the long-term preservation of axotomized neurons, when treated with the 31 kD FGF2 expressing hESCs. The results demonstrated preservation of motoneurons in all cell therapy-treated groups, with the most significant effect observed in the group treated with Dox-induced hESCs (73% ± 2%), compared to the reimplantation-only group (34% ± 2%) (AvR + HFB + Dox + hESC 31kD vs. AvR + HFB + Dox, ****p < 0.0001). A statistically significant difference was also noted in comparison to the groups treated with non-activated (55% ± 2%) and adult mesenchymal stem cells (55% ± 2%) (AvR + HFB + hESC 31kD OFF and AvR + HFB + Dox + hMSC vs. AvR + HFB + Dox, ***p = 0.0001). Furthermore, the induced hESC treatment demonstrated greater neuroprotection than any other cell treatment (AvR + HFB + Dox + hESC 31kD vs. AvR + HFB + hESC 31kD OFF and AvR + HFB + Dox + hMSC, *p = 0.02) (Supplementary Fig. 3).

GFAP expression, an indicator of astrocytic reactivity, increased in all groups during the second-week post-injury on the ipsilateral side compared to the contralateral, with an average increase of 189%. The highest reactivity was observed in the group that underwent root reimplantation alone (AvR + HFB + Dox, 258% ± 17%). Additionally, significant differences were observed when compared to groups receiving other treatments combined with root repair, such as commercial FGF2 (142% ± 4%), hMSCs (160% ± 12%), Dox-induced hESCs (179% ± 7%), and hESCs OFF (207% ± 11%) (AvR + HFB + Dox vs. AvR + HFB + Dox + Commercial FGF2, AvR + HFB + Dox + hMSC, AvR + HFB + Dox + hESC 31 kD, and AvR + HFB + hESC 31 kD OFF, ****p < 0.0001, ****p < 0.0001, ***p = 0.0008, and *p = 0.03, respectively). The hESC OFF-treated group also exhibited a significant difference when compared to the commercial FGF2-treated group (AvR + HFB + hESC 31 kD OFF vs. AvR + HFB + Dox + Commercial FGF2, **p = 0.005).

**Fig. 2:**
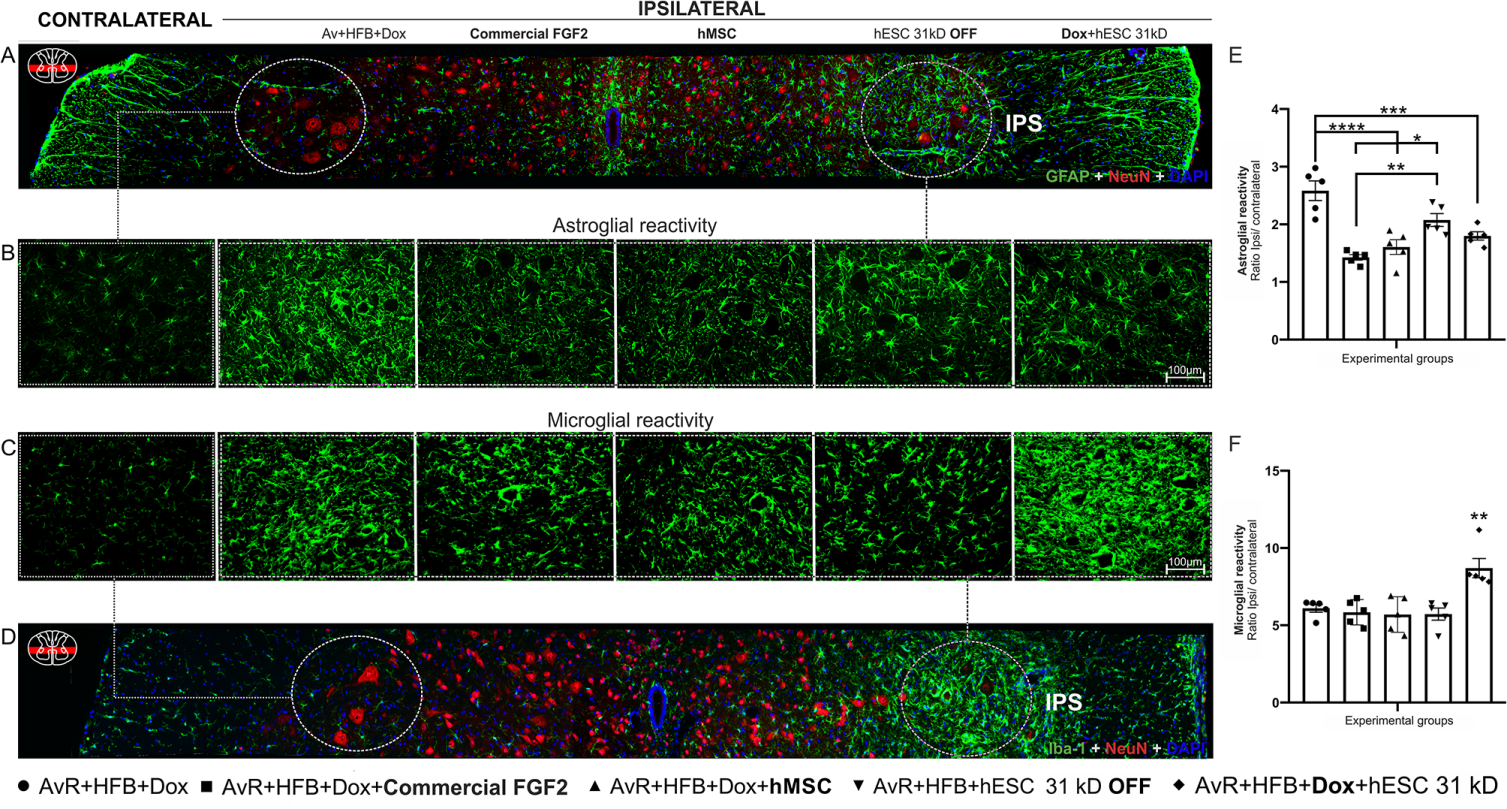
Assessment of glial modulation by 31 kD hESCs following root avulsion and reimplantation. Representative images of (A - B) astroglial and (C – D) microglial reactivity. (E) Quantification of astroglial reactivity. All groups exhibited reduced astrocytic expression. (F) Quantification of microglial reactivity. Observe the increased microgliosis in the group treated with activated hESC (AvR + HFB + Dox + hESC 31 kD) (**p < 0.01). The astroglial and microglial reactivities were assessed using the integrated density of pixels (ipsilateral/contralateral ratio, *n* = 5 per group). Data are presented as mean ± SEM; * *p* < 0.05; ** *p* < 0.01; *** *p* < 0.001; **** *p* < 0.0001

Microglial response to injury was also observed. Of note, the DOX-induced hESC-treated group (AvR + HFB + Dox + hESC 31 kD) showed a statistically significant upregulation compared to other treatments (**p < 0.01). The mean values and standard error of the mean ratios are summarized in Table 4 and illustrated in Fig. 2.

**Table 4:** Groups from the second set of experiments (avulsion + reimplantation) - data related to glial reactivity

Groups	Astroglial reactivity		Microglial reactivity	
	Mean	SEM	Mean	SEM
AvR + HFB + Dox	2.58	0.17	6.10	0.25
AvR + HFB + Dox + **hMSC**	1.61	0.13	5.70	0.51
AvR + HFB + hESC 31 kD **OFF**	2.08	0.11	5.72	0.39
AvR + HFB + **Dox** + hESC 31 kD	1.80	0.07	8.70	0.62

Astroglial and microglial reactivity (2 weeks) following root reimplantation in the different experimental groups. Mean ± SEM

#### Preservation of inhibitory inputs combined with reduced glutamatergic innervation following 31 kD hESCs therapy

Two weeks after root injury and repair using HFB, synaptic coverage was evaluated by immunohistochemistry. The neurorrhaphy-only group displayed the most significant synaptic detachment (40% ± 4%), whereas the doxycycline-activated hESC group demonstrated the highest preservation of synaptic coverage (79% ± 4%), followed by treatments with hMSC (62% ± 2%), non-activated hESC (64% ± 1%), and commercial FGF2 (49% ± 4%). Commercial FGF2 treatment was the only one without a statistically significant difference compared to the control group (AvR + HFB + Dox vs. AvR + HFB + Dox + hESC 31 kD, AvR + HFB + Dox + hMSC, and AvR + HFB + hESC 31 kD OFF, ****p < 0.0001, **p = 0.003, and **p < 0.01, respectively). Groups treated with commercial FGF2 and hMSC also showed a significant difference compared to the treatment with the highest synaptic coverage (AvR + HFB + Dox + Commercial FGF2 and AvR + HFB + Dox + hMSC vs. AvR + HFB + Dox + hESC 31 kD, ***p = 0.0002 and *p = 0.03).

The Dox-induced 31 kD hESC-treated group displayed the lowest glutamatergic coverage compared to animals receiving only root reimplantation (AvR + HFB + Dox + hESC 31kD vs. AvR + HFB + Dox, 48% ± 5% and 71% ± 7%, respectively; *p = 0.03). Although other groups also exhibited decreased VGLUT1 immunostaining, no statistical significance was observed among the groups.

**Fig. 3:**
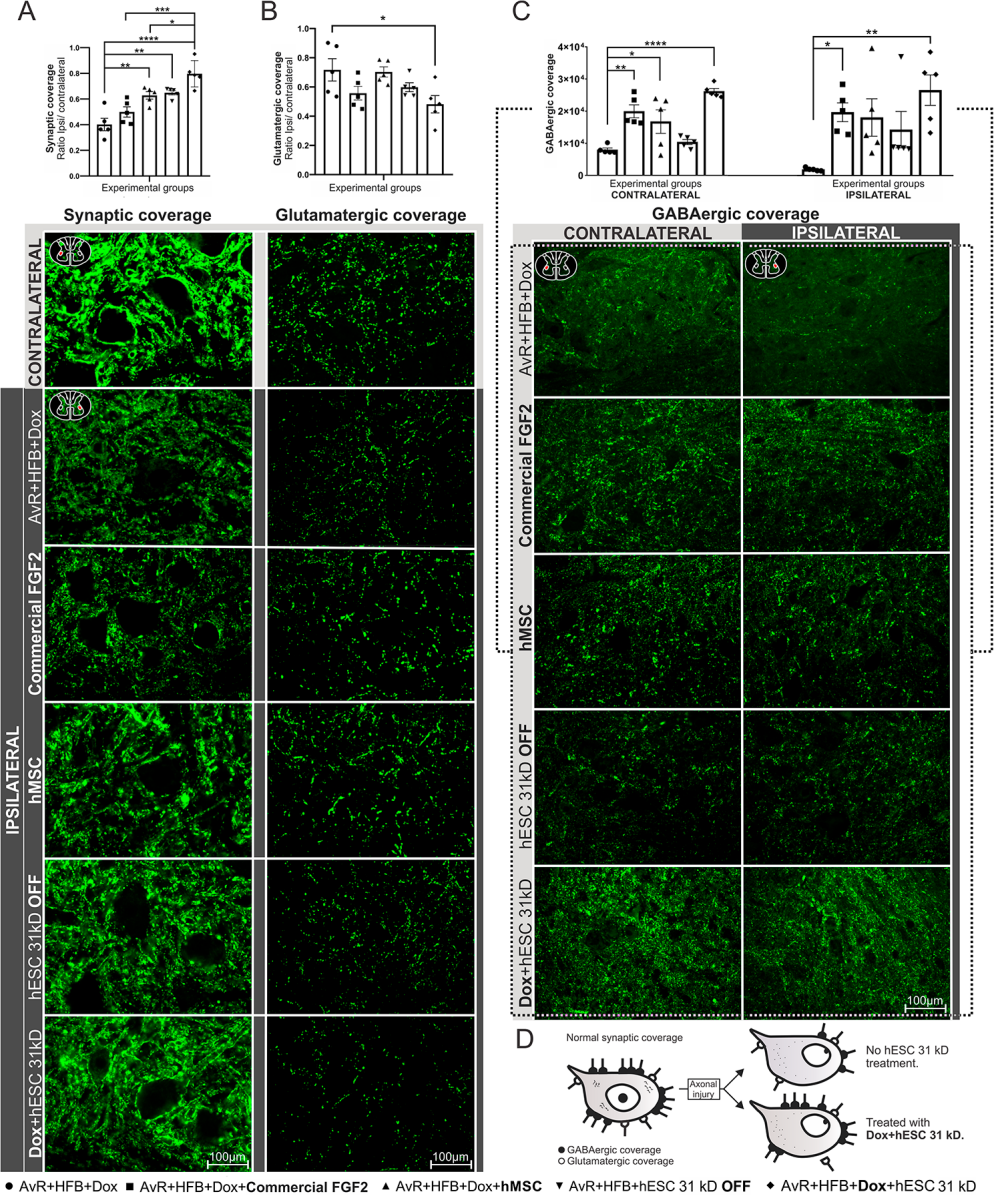
Modulation of synaptic coverage and glutamatergic and GABAergic expression by 31 kD hESC treatment. (A) Approximately 80% of synaptic coverage was preserved with the treatment involving activated hESCs (AvR + HBF + Dox + hESC 31 kD). (B) glutamatergic coverage was reduced (*p < 0.05), while (C) GABAergic coverage was preserved, with the latter effect observed both ipsi- and contralateral to the lesion. (D) Schematic view of the preservation of synapses, after treatment with 31kD hESCs, favoring GABAergic (inhibitory) over excitatory (glutamatergic) inputs. Quantification of synaptic coverage, glutamatergic, and GABAergic coverage using the integrated density of pixels (ipsilateral/contralateral ratio, *n* = 5 per group). Data are presented as mean ± SEM; *p < 0.05; **p < 0.01; ***p < 0.001; ****p < 0.0001

GABAergic coverage was affected on both the ipsilateral and contralateral sides of the lesion. On the contralateral side, the doxycycline-induced hESC-treated group exhibited the highest GABAergic coverage, followed by groups treated with commercial FGF2 and hMSC (AvR + HFB + Dox + hESC 31 kD vs. AvR + HFB + Dox, ****p < 0.0001; AvR + HFB + Dox + Commercial FGF2 vs. AvR + HFB + Dox, **p = 0.001; AvR + HFB + Dox + hMSC vs. AvR + HFB + Dox, *p < 0.05). On the ipsilateral side, both the induced hESC-treated group and the commercial FGF2-treated group showed significantly greater values (AvR + HFB + Dox + hESC 31kD vs. AvR + HFB + Dox, **p = 0.002; AvR + HFB + Dox + Commercial FGF2 vs. AvR + HFB + Dox, *p = 0.03). The mean and standard error of the mean ratios are summarized in Table 5; Fig. 3.

**Table 5:** Groups from the second set of experiments (avulsion + reimplantation) - data related to synapse coverage

Groups	Synaptic Coverage		Glutamatergic coverage		GABAergic coverage			
					Contralateral		Ipsilateral	
	Mean	SEM	Mean	SEM	Mean	SEM	Mean	SEM
AvR + HFB + Dox	0.40	0.05	0.72	0.08	7956.00	565.00	1739.00	196.30
AvR + HFB + Dox + **Commercial FGF2**	0.50	0.04	0.56	0.05	19932.00	2021.00	17376.00	2670.00
AvR + HFB + Dox + **hMSC**	0.63	0.03	0.70	0.03	16742.00	3649.00	16485.00	5329.00
AvR + HFB + hESC 31 kD **OFF**	0.65	0.02	0.60	0.03	10377.00	779.30	13029.00	5175.00
AvR + HFB + **Dox** + hESC 31 kD	0.80	0.05	0.48	0.06	26176.00	880.90	24248.00	4329.00

Glutamatergic and GABAergic coverage in the different experimental conditions post root reimplantation, 2 weeks after injury and repair. Mean ± SEM

#### Gene expression after 31kD hESCs therapy

qRT-PCR was performed two weeks after injury/repair of the ventral roots. Non lesioned rats were used as the control group. The evaluation of relative gene expression focused on anti-inflammatory cytokines (TGFβ and IL10), pro-inflammatory cytokines (IL1β, TNFα, and IL6), neurotrophic factors (VEGF, FGF2, HGF, BDNF, and GDNF), and MHC-I-related molecules (β2m and CD3ζ), as shown in Table 2.

TGFβ expression was significantly higher in the hMSC-treated group compared to animals that received reimplantation alone or those without injury (AvR + HFB + Dox + hMSC vs. AvR + HFB + Dox and no injury, *p < 0.05 and ***p < 0.001). There was also a statistically significant increase in the induced human embryonic stem cell-treated group (hESCs 31 kD) compared to animals without injury (AvR + HFB + Dox + hESC 31 kD vs. no injury, *p < 0.05). IL10 expression, on the other hand, was upregulated only in the hMSC-treated group (*p < 0.05).

The relative expression of IL1β and TNFα genes was similar, increasing significantly only in the hMSC-treated group (**p < 0.01). As expected, the expression of IL6 in the group without injury was lower than in all other groups (no injury vs. AvR + HFB + Dox, AvR + HFB + Dox + hMSC, AvR + HFB + hESC 31 kD OFF, and AvR + HFB + Dox + hESC 31 kD, ***p < 0.001).

Genes related to neurotrophic factors showed no significant difference, except for HGF, where an increase in relative expression was observed in the group treated with hMSC.

**Fig. 4:**
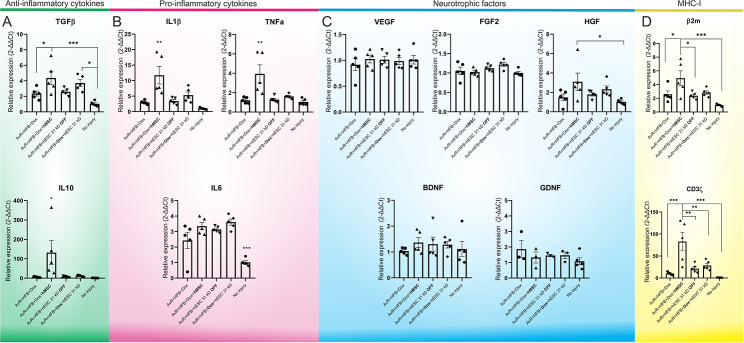
Relative gene expression by qRT-PCR. (A) anti-inflammatory cytokines (TGFβ and IL10), (B) pro-inflammatory cytokines (IL1β, TNFα, and IL6), (C) neurotrophic factors (VEGF, FGF2, HGF, BDNF, and GDNF) and (D) genes related to the MHC I immune response (β2m and CD3ζ). It is important to note that although hESCs have a slight modulation on TGFβ, they do not indiscriminately induce the production of pro-inflammatory molecules, as with hMSCs, such as IL1β and TNFα. Mean + SEM; *p < 0.05; **p < 0.01; ***p < 0.001

Although the engraftment of xenogenic stem cells might result in immune response, the hESCs displayed significantly lower levels of MHC I related gene transcripts in comparison to the hMSCs counterpart. Of note, the reimplantation of the avulsed roots, in combination with HFB did not contribute to an important upregulation of B2m as well as CD3ζ (Fig. 4).

### Functional recovery

#### Improvement of temporal gait-related parameters (stand phase, swing phase, and gait cycle)

**Fig. 5:**
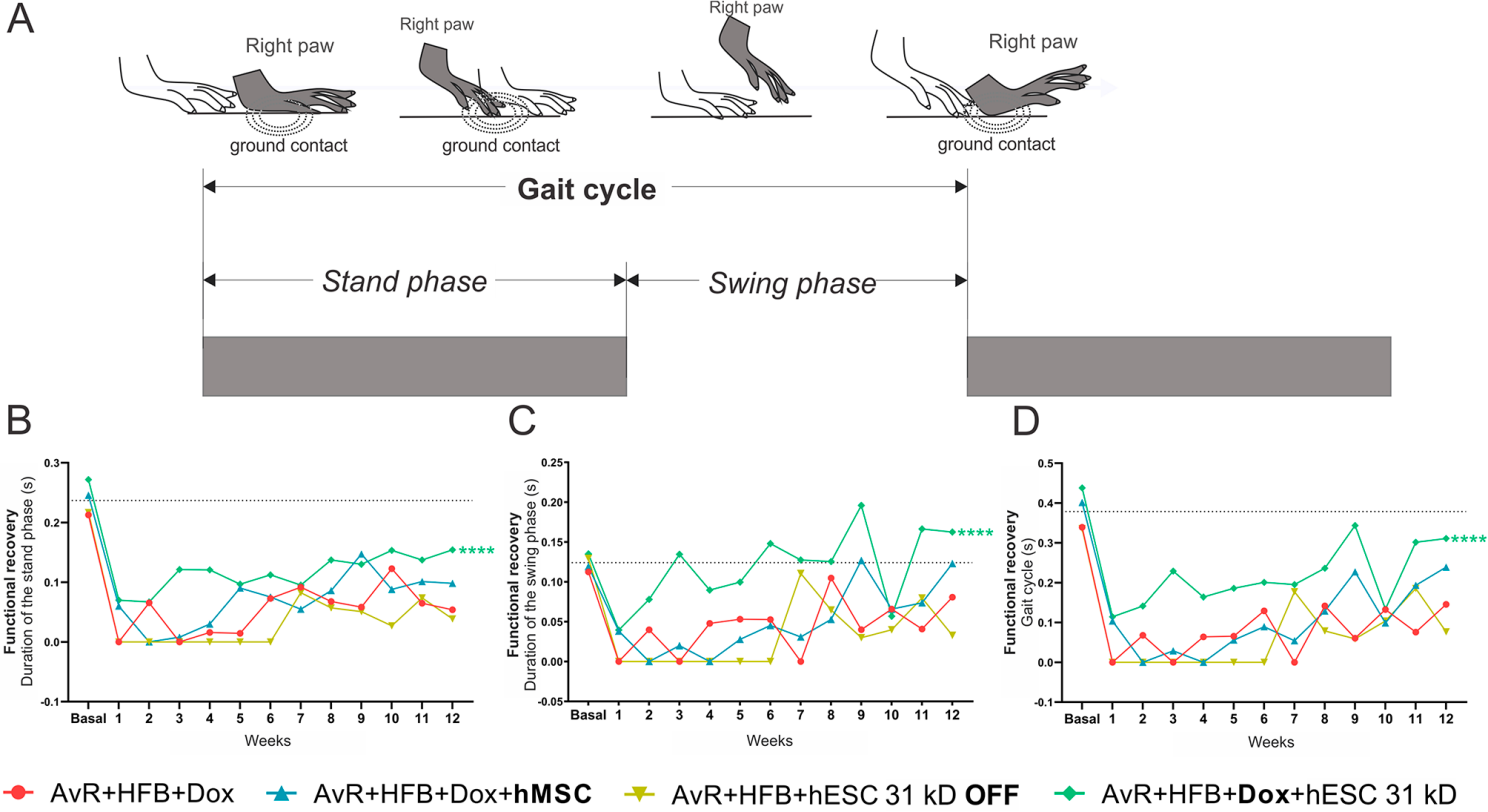
Analysis of gait recovery using the CatWalk system (walking track test). (A) Schematic drawing illustrating the different phases of the gait cycle, such as stand and swing phases. (B) Animals treated with activated hESCs (AvR + HFB + Dox + hESC 31 kD) showed a significant improvement (****p < 0.0001) in the duration, in seconds, of foot contact with the floor (stand phase). (C) At the end of the analysis of the swing phase, the animals treated with hMSC showed behavior similar to the baseline, whereas the group treated with activated hESC (AvR + HFB + Dox + hESC 31 kD) significantly prolonged the swing phase when compared to the group submitted only to reimplantation (AvR + HFB + Dox). (D) For the gait cycle, in seconds, the animals treated with activated hESC (AvR + HFB + Dox + hESC 31 kD) are those that are closest to the preoperative period and the only ones to present a significant difference when compared to the group that received only the reimplantation (AvR + HFB + Dox). Mean + SEM. ****p < 0.0001

Gait-related temporal parameters were acquired over a period of twelve weeks using the Catwalk system (Fig. 5). The gait cycle is composed of the stand phase and the swing phase, the first being the period in which a given paw is in contact with the ground and the second, the interval it spends in the air, as shown in a visual diagram (Fig. 5A).

The duration of the stand phase is measured in seconds. The baseline value refers to the pre-injury period (x̄ = 0.24s, where x-bar is the mean value of all animals at that time point). It is observed that, soon after the avulsion of the ventral roots, the animals start to neglect the paw ipsilateral to the nervous lesion (x̄ = 0.03 s, in the first week). Over the twelve weeks of experimentation, all groups showed a non-significant recovery of this parameter, except for the group treated with hESC 31kD (AvR + HFB + Dox + hESC 31 kD x AvR + HFB + Dox, ****p < 0.0001) (Fig. 5B).

The swing phase, referring to the interval in seconds during ambulation when the paw is not in contact with the glass platform between two consecutive support phases, presented similar values for all groups before the injury (x̄ = 0.12 s, dashed line in Fig. 5C). In the first week after avulsion, the minimum value is reached (x̄ = 0.01 s), which improves over the subsequent twelve weeks. However, only the group treated with activated hESCs showed a significant difference as compared to the animals submitted only to reimplantation (AvR + HFB + Dox + hESC 31 kD x AvR + HFB + Dox, ****p < 0.0001) (Fig. 5C).

**Fig. 6:**
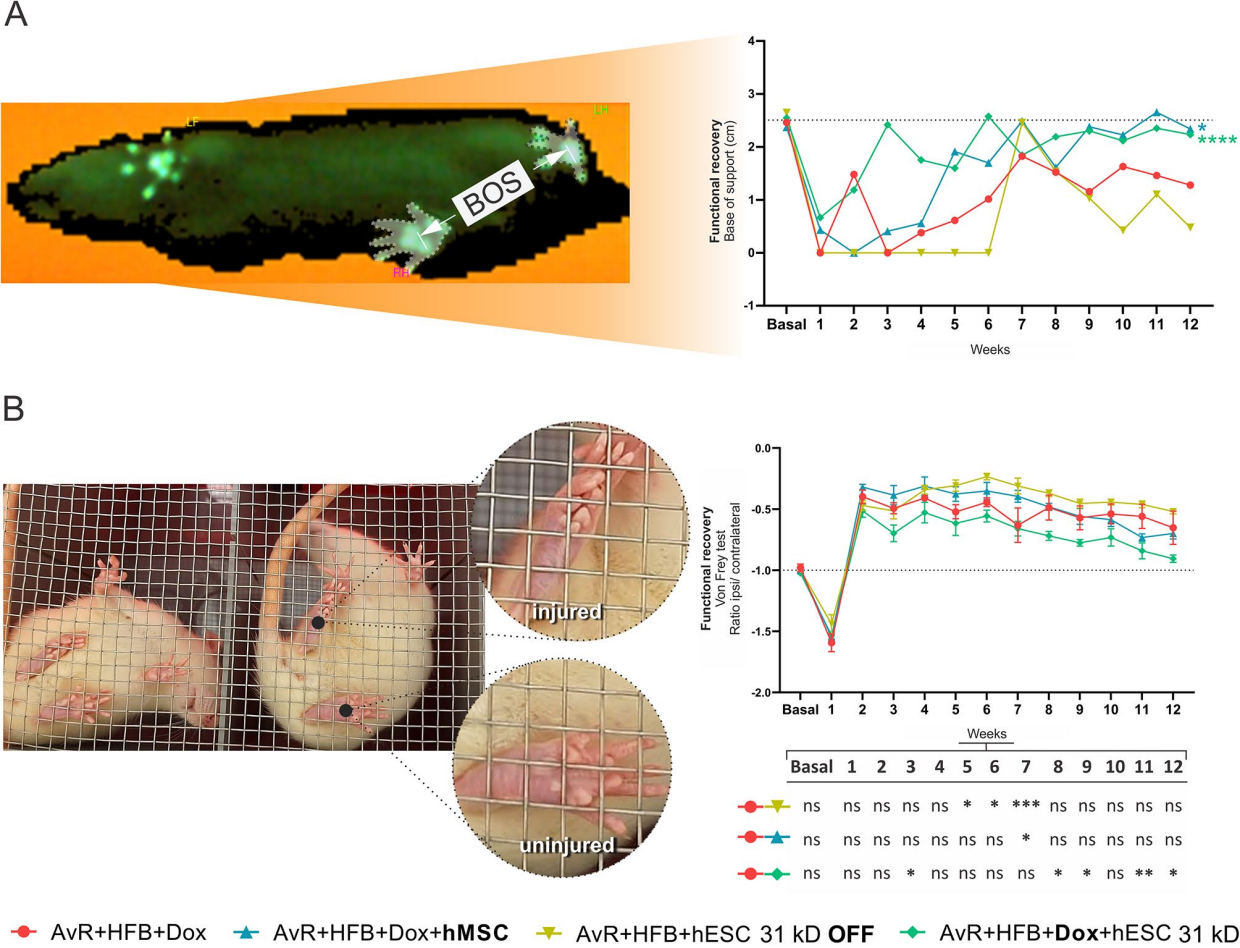
31 kD FGF2 - hESC improves motor coordination parameters and nociceptive threshold. (A) Graph of BOS values, acquired longitudinally, starting from the pre-injury condition (baseline). All groups show some ascendance to the right of the graph, towards the baseline; however, only the groups treated with hMSC and activated hESC showed significance when compared with the group that received only reimplantation (AvR + HFB + Dox + hMSC and AvR + HFB + Dox + hESC 31 kD x AvR + HFB + Dox, *p < 0.05 and ****p < 0.0001, respectively). (B) Representative image of the bottom view of the Von Frey system, highlighting the difference in the morphology of the plantar surface of the injured and uninjured paws. The graph shows the elimination of the withdrawal reflex in the first week, followed by an earlier response than that found in non-injury conditions. It is noted that the recovery of all groups follows in parallel; however, in the table, it is possible to observe that the group treated with hESCs is the first to show some significant difference in relation to the group that did not receive cell therapy (AvR + HFB + Dox + hESC 31 kD x AvR + HFB + Dox, day 3 after injury, *p < 0.05) and also the only one to maintain some difference later (weeks 11 and 12, **p < 0.01 and *p < 0.05, respectively). AvR: Avulsion and reimplantation; HFB: Heterologous fibrin biopolymer; hESC: Human embryonic stem cell; Dox: Doxycycline; kD: Kilodalton (atomic mass unit). Mean + SEM; *p < 0.05; **p < 0.01; ***p < 0.001; ****p < 0.0001

The gait cycle, which is the sum of the stand phase and the swing phase and represents the time in seconds between two consecutive contacts of the same paw, is homogeneous between the groups before the injury (x̄ = 0.38 s, dashed line in Fig. 6E). One week after the injury, the cycle tends to zero due to paralysis of the limb (x̄ = 0.05 s). Gradually, the animals recover, but those treated with activated hESCs are the closest to the preoperative gait cycle and present a significant difference compared to the group that received only the reimplantation (AvR + HFB + Dox + hESC 31 kD x AvR + HFB + Dox, ****p < 0.0001) (Fig. 5D).

#### Improvement of gait-related spatial parameters (PFI, maximum contact area, and maximum intensity relativized by the stand phase)

**Fig. 7:**
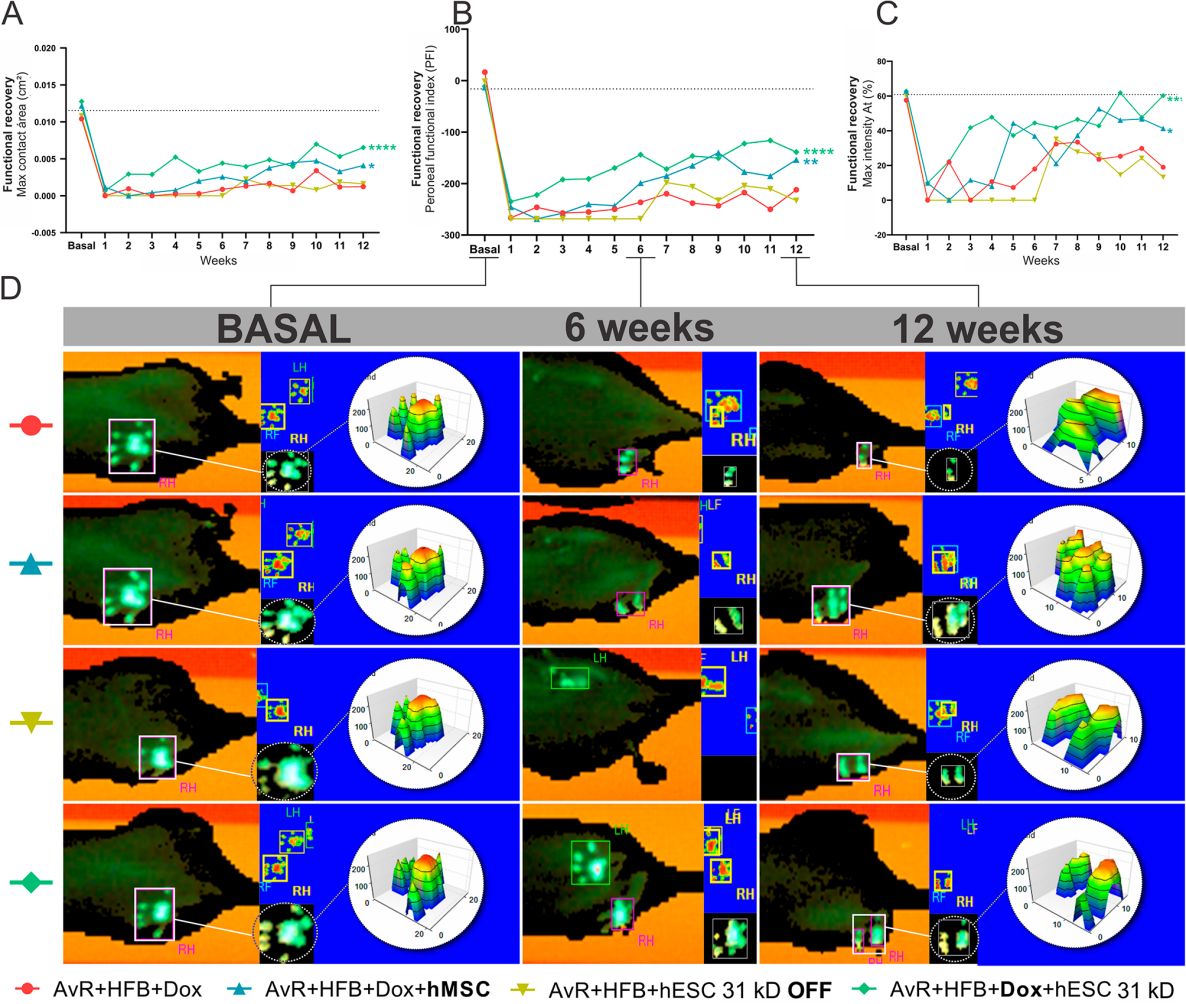
31 kD FGF2 - hESC improves gait temporal parameters. Gait progression is observed through (A) maximum contact area and (B) PFI, in groups of animals submitted to different treatments. It is notable that animals treated with activated hESC and mesenchymal cells (AvR + HFB + Dx + hESC 31 kD and AvR + HFB + hMSC) performed better with the use of the plantar surface during gait. (C) Indirect measurement of pressure (intensity) relativized by stand phase. Animals treated with hMSC and hESC (AvR + HFB + Dox + hMSC and AvR + HFB + Dox + hESC 31 kD) showed statistically significant improvement (*p < 0.05 and ****p < 0.0001, respectively). (D) Representative panel of images obtained using the CatWalk system at three different time points (baseline, 6 weeks, and 12 weeks). An inferior view of the body and the plantar surface of the animals is shown, in addition to the representation of the latter in a three-dimensional graph (intensity + contact area). Mean + SEM. *p < 0.05; **p < 0.01; ***p < 0.001; ****p < 0.0001

The peroneal functional index (PFI) is a parameter that considers the width and length of the animals’ footprints during locomotion, while the maximum contact is the measurement of the area in which the weight is distributed during the stand phase (Fig. 7). In the analysis of the PFI after avulsion of the ventral roots, a decrease in the index to values below − 200 was observed, followed by fluctuations in that index during the twelve-week evaluation period. The group that received root replantation alone (AvR + HFB + Dox) and the one treated with silent embryonic stem cells (AvR + HFB + hESC 31 kD OFF) did not show significant improvement over time. In contrast, animals treated with mesenchymal stem cells exhibited a significant recovery of PFI (AvR + HFB + Dox + hMSC x AvR + HFB + Dox, **p < 0.01), which was significantly enhanced in the group treated with activated hESCs (AvR + HFB + Dox + hESC 31kD x AvR + HFB + Dox, ****p < 0.0001).

Analysis of the maximum contact area followed a similar pattern, with an immediate absence of the injured paw’s record after injury and sporadic recordings in the following weeks. The groups that received only reimplantation and treatment with non-activated cells (AvR + HFB + Dox and AvR + HFB + hESC 31 kD OFF) did not show significant improvement. Conversely, both the group treated with hMSCs and the one that received activated hESCs demonstrated a significant recovery of the plantar surface impression at the end of the evaluation (AvR + HFB + Dox + hMSC and AvR + HFB + Dox + hESC 31 kD x AvR + HFB + Dox, *p < 0.05 and ****p < 0.0001, respectively).

The maximum intensity relates to the stance phase, expressed as a percentage, representing the greatest weight-bearing exerted during gait to the total time of paw contact on the glass platform. This measurement reveals that the animals in the groups submitted only to reimplantation and those treated with non-activated hESC (AvR + HFB + Dox and AvR + HFB + hESC 31 kD OFF) maintained low weight-bearing on the injured paw until the twelfth week (x̅= 19% and 13%, where x̅ is the average of the values, of each group mentioned above, respectively). In contrast, the group treated with hMSC (x̅= 41%) showed significant improvement (AvR + HFB + Dox + hMSC x AvR + HFB + Dox, *p < 0.05), and the improvement in the group with doxycycline-activated hESC (x̅= 60%) significantly better (AvR + HFB + Dox + hESC 31 kD x AvR + HFB + Dox, ****p < 0.0001).

Footprint images obtained by the software reveal that before the injury (baseline), the outline of the plantar region and the rats’ toes are well-defined. Following avulsion, due to the lack of use of the injured paw, there is minimal marking during the first weeks of walking. By the sixth week of evaluation, the differences in recovery between the groups become apparent. By the twelfth week of evaluation, it is noticeable that the animals treated with activated hESCs exhibit a paw and toe contour closer to that seen in the non-injured condition, although the digits are not yet completely separated. Animals treated with reimplantation alone demonstrate only the beginning of weight-bearing on the sole in the region of the distal phalanges. In the three-dimensional graphic representation of the right paw (injured), both in the baseline condition and at the end of the twelfth week, the distribution of the intensity exerted on the platform on an absolute scale from 0 to 200 reaches the maximum value (red) in all groups.

#### Improvement of motor coordination parameters (BOS) and nociceptive threshold

The base of support is a measurement of coordination and balance that utilizes the distance between the hind limbs during locomotion (Fig. 6A). Before the injury, all groups had similar measurements (x̄ = 0.25, dashed line). After avulsion, values drop close to zero in all groups (x̄ = 0.03, dashed line) and follow different recovery patterns. At the end of twelve weeks, both the group treated with hMSCs and the one treated with 31 kD hESCs activated with Dox reached values close to the baseline, being statistically significant when compared with the group that received reimplantation alone (AvR + HFB + Dox + hMSC and AvR + HFB + Dox + hESC 31 kD x AvR + HFB + Dox, *p < 0.05 and ****p < 0.0001, respectively).

To assess the nociceptive threshold (Fig. 6B), a von Frey electronic system was employed to apply a stimulus to the plantar region of the animals in order to elicit the withdrawal reflex. It was observed that compared to animals that received only avulsion with reimplantation, animals treated with activated hESCs demonstrated a significant and consistent improvement in the last two weeks of evaluation (AvR + HFB + Dox x AvR + HFB + Dox + hESC 31 kD at the 11th and 12th weeks, **p < 0.01 and *p < 0.01, respectively). None of the other groups showed a statistically significant difference in the last week, compared to the group without cell treatment (AvR + HFB + Dox). Of note, the treatment with non-activated hESCs displayed an antagonistic behavior to the activated cells, with the animals exhibiting greater sensitivity to the plantar stimulus from the fifth week to the seventh week after the injury.

## Discussion

Spinal root injuries, which account for 2–5% of all trauma cases in the United States, are frequently caused by assaults or accidents involving motor vehicles [52]. In cases of complete damage to the root filaments, such as sectioning or avulsion, surgical repair is necessary [53, 54]. Nevertheless, CNS/PNS reconnection is not sufficient to assure significant sensorimotor recovery. Thus, the combination of different approaches is required, and pre-clinical studies are of fundamental importance to evaluate the neurobiology of the regenerative process.

Pharmacological treatments, stem cell therapy, and trophic factors, such as FGF2, are being considered as combined treatment options with neurorrhaphy/root coaptation [43, 55–57]. We demonstrated [31] that bioengineered hESCs, which overexpress FGF2 (18kD isoform) rescued 60% of avulsed motoneurons. In the current study, we investigated the effectiveness of hESCs expressing 3 different isoforms of FGF2 (18, 23, and 31 kD) to identify the isoform with the greatest potential for maintaining synaptic coverage, glial modulation, and, most importantly, enhanced neuroprotective capacity. Our results indicate that hESCs with overexpression of the highest molecular weight FGF2 (31 kD) stand out as the most beneficial in terms of neuroprotection and preservation of inputs to the injured motoneurons This was confirmed in the long-term experiments that included the root reimplantation with HFB, where the rescue of motoneurons was close to 80% by twelve weeks post-injury, combined with robust network preservation at the motor nucleus of the spinal cord (lamina IX of Rexed). Such results were significantly better in comparison to our previous work with the 18 kD isoform [31].

The preservation of axotomized motoneurons may be associated with a significant reduction in reactive astrogliosis. Such downregulation seems relevant to the preferential preservation of inhibitory presynaptic inputs over the excitatory counterparts, reducing excitotoxicity by glutamate [58]. Another essential component in the maintenance of synaptic coverage is the microglial reactivity as they contribute to synaptic pruning and debris clearance.

Araújo et al., (2017) [31] found, in the motor root avulsion model using HFB as a scaffold, that the group treated with 18 kD hESCs showed half of the astroglial reactivity as compared to the control group. In the present results, the 31 kD isoform demonstrates an even more promising outcome, since reactive astrogliosis was downregulated by 60%. Such a decrease indicates a lower probability of glial scar formation in the gray/white matter interface, which is considered critical for the regrowth of the sectioned axons towards the reimplanted roots [59–61]. One possible explanation for such a decrease in astroglial response is the presence of FGF receptors (FGFR) in such cells. Kang et al., (2014) [62] demonstrated that deletion of FGFR results in intense astrogliosis. Of importance, however, is not to completely suppress the astroglial reactivity, as persistent motor deficits after mild or moderate spinal cord injury may take place [63]. In this sense, the findings herein support the modulation of astrogliosis in the group treated with activated embryonic stem cells (AvR + HFB + Dox + hESC 31 kD) as a positive indicator, since it translated into better functional recovery.

Microglia sense and regulate neuronal activity through the surveillance of the environment. Microglial processes establish rapid contact with various CNS cells, such as neurons (including presynaptic and postsynaptic elements), perivascular astroglial cells, and other structures present in the neuronal milieu [64]. Our results show that 31 kD hESC engraftment (AvR + HFB + Dox + hESC 31 kD), leads to highly activated microglia with an increase of approximately 78.1% of Iba-1 labeling. This has already been observed by Araújo et al., (2017) [31] after the use of the 18 kD isoform, indicating that such experimental approach results in robust microglial response. Based on the overall positive morphological and behavioral outcome, it is possible that such activation contributed to the regenerative response by accelerating debris phagocytosis. Microglial cells can acquire a pro-regenerative profile that is beneficial to tissue repair [65–67]. Nevertheless, the qRT-PCR, two weeks after the injury, did not provide evidence of growth factors or pro-inflammatory cytokines upregulation, although it might have happened at earlier time points. Thus, further evaluations are necessary to understand the role of microglial activation in response to hESCs therapy.

In the context of response to injury, the control of excitatory neurotransmitter release is of particular importance. The exacerbated increase of glutamate, a neurotransmitter recognized for its excitatory function, is associated with cellular excitotoxicity. Such over-activation results in intracellular alterations, including the production of reactive oxygen and nitrogen species (ROS and NOS), as demonstrated by Sadowsky et al. (2002) [68]. Along with the inflammatory process, such events culminate in significant neuron death and exacerbate motor deficits [63]. Thus, the balance of glutamatergic stimuli in injured motoneurons may represent a favorable neuroprotective strategy [69]. In this context, the present study provided evidence that 31 kD FGF2 - hESCs avoided exacerbation of the number of glutamatergic synapses.

In addition to excitatory balance, we evaluated γ-aminobutyric acid (GABA) inputs, representing inhibitory synapses [70]. GABAergic coverage increased significantly in all groups analyzed compared to the reimplantation alone. Interestingly, such upregulation was found both on the contralateral and ipsilateral sides of the lesion. The balance of inhibitory and excitatory terminals after injury may play a crucial role after injury [21, 71]. Accordingly, such readjustment is particularly important for motor recovery and reduction of neuropathic pain [72].

The positive morphological results mentioned above are in line with the functional recovery after root reimplantation and 31 kD FGF2 - hESC therapy. Although recuperation was not complete with cell therapy, animals that received reimplantation alone showed almost no regain of function after the 12-week evaluation. Contrarily, animals treated with stem cells expressing 31 kD FGF2 performed better on several gait parameters. In human patients, gait independence is related not only to ambulation itself but also to a better quality of life [73, 74]. The recovery observed by the analysis of the nociceptive threshold by the von Frey system was also noticeable since the rats treated with the induced hESCs (31 kD) performed better and displayed close to normal withdrawal reflex at the experiment endpoint.

Relative gene expression showed that both hESCs and hMSCs produce anti-inflammatory cytokines, such as TGFβ, whose upregulation in the CNS has been described after injuries and neurodegenerative diseases [75, 76]. It is important to emphasize that, although our study did not identify differences in relation to neurotrophic factors (VEGF, FGF2, BDNF, and GDNF), it corroborates the literature regarding the production of HGF by hMSCs [77]. However, unlike hMSCs, 31 kD FGF2 - hESCs did not express pro-inflammatory cytokines such as IL1β and TNFα, which reinforces the hypothesis that better functional recovery may be the result of more efficient local immunomodulation.

Confocal microscopy showed that the higher molecular weight isoforms of FGF2 tend to be concentrated more in the nucleoli, which might be related to more active involvement in the regulation of gene expression. It may indicate an autocrine stimulation of the hESCs themselves, in line with the published data [34]. However, in other experiments, we observed an enhanced general neurotrophic role of the HMW FGF2. On the other hand, the qRT-PCR data did not show any significant enhancement of neurotrophin mRNA in the spinal cord microenvironment. The precise mechanism of the observed neuroprotection of the FGF2 overexpressing hESC is not yet known. It presumes either direct delivery of the FGF2 or action via activation of expression of various neurotrophic factors. Our data showed that the overexpressed FGF2 localized predominantly in nuclei and less so in the cytoplasm. The function of extracellular HMW FGF2 has not been well established in the nervous system, while both LMW and HMW were shown to have protective effects [78]. FGF2 primarily regulates gene expression by binding to its receptors on the surface of the target cells. This binding activates several signaling pathways, including the mitogen-activated protein kinase (MAPK), phosphoinositide-3 kinase (PI3K), and protein kinase C (PKC) pathways, which ultimately lead to the activation or repression of specific genes. FGF2 is known to activate the transcription factor c-Fos, which in turn activates the expression of several genes, including those involved in cellular proliferation, differentiation, and survival. FGF2 also regulates the expression of genes encoding extracellular matrix proteins such as collagen and fibronectin, which are important for cell migration and tissue repair [79, 80]. FGF2 can also directly regulate the activity of certain transcription factors. Additionally, FGF2 is related to the activity of epigenetic regulators, such as histone deacetylases [81].

Regenerative properties of the various isoforms of FGF2 are actively investigated. Thus, the elimination of HMW FGF2 was shown to be cardioprotective in knockout mice [82]. Similarly, the loss of HMW FGF2 offered protection in the model of osteoarthritis in a similar knockout model [83]. Overexpression of HMW FGF2 resulted in decreased dentin and alveolar bone mineralization in transgenic mice [84]. These, and our results suggest that protective and regenerative roles of both LMW and HMW FGF2 are highly dependent on the context of the experimental setup, while both LMW and HMW FGF2 can display neuroprotective and immunomodulatory roles after engraftment of FGF2 overexpressing stem cells.

## Conclusion

In conclusion, hESC altered by bioengineering for overexpression of 31 kD FGF2 display neuroprotective properties, even in the worst possible context, where there was no reimplantation (1st set of experiments). Therapy with activated hESCs not only preserved motoneurons but also provided stability in the neuronal circuitry, maintaining synaptic coverage and favoring inhibitory synaptic inputs, mitigating the sprouting of glutamatergic boutons. In addition to beneficial effects at the neuronal level, 31 kD FGF2 - hESC also modulated the glial response, particularly by controlling reactive astrogliosis. Although microgliosis can be interpreted as an exacerbated pro-inflammatory response, the phenotype of these cells was not evaluated in this study, which may in turn be investigated in future analyses. However, considering the benefits observed in all parameters related to functional recovery and reduced expression of pro-inflammatory cytokines, it is plausible to assume that such microglia activation was not detrimental.

Future research is necessary to further understand the molecular and cellular mechanisms involved in neuroprotection and functional recovery provided by 31 kD FGF2 - hESC therapy. Nevertheless, the present data reinforce that stem cell therapy in combination with effective surgical approaches may provide the grounds for successful repair of spinal cord injuries, in particular those involving plexus lesions.

## Electronic supplementary material

Below is the link to the electronic supplementary material.

Supplementary Material 1

## Data Availability

All relevant data and materials supporting our findings are available upon request.

## Abbreviations

BDNF: Brain-Derived Neurotrophic Factor
CNS: Central Nervous System
FGF2: Fibroblast Growth Factor 2
GABA: Gamma-Aminobutyric Acid
GDNF: Glial Cell Line-Derived Neurotrophic Factor
HFB: Heterologous Fibrin Biopolymer
HGF: Hepatocyte Growth Factor
HMW: High Molecular Weight
hESCs: Human Embryonic Stem Cells
LMW: Low Molecular Weight
MEF: Murine Embryonic Fibroblast
MHC-I: Major Histocompatibility Complex Class I
MAPK: Mitogen-Activated Protein Kinase
NOS: Nitric Oxide Synthase
PNS: Peripheral Nervous System
PI3K: Phosphoinositide-3 Kinase
PKC: Protein Kinase C
qRT-PCR: Quantitative Real Time Polymerase Chain Reaction
ROS: Reactive Oxygen Species
VEGF: Vascular Endothelial Growth Factor
