# Supplementary material for: Embryonic stem cells overexpressing high molecular weight FGF2 isoform enhance recovery of pre-ganglionic spinal root lesion in combination with fibrin biopolymer mediated root repair
Source: Stem Cell Res Ther. 2024 Mar 5;15:63. doi: 10.1186/s13287-024-03676-6 (PMC10913678; doi:10.1186/s13287-024-03676-6)
Supplement: Supplementary file 1 — Supplementary Material 1 [file 13287_2024_3676_MOESM1_ESM.docx]

Supplementary Table 1

**Supplementary Table 1.** Primary and secondary antibodies used in the study.

|  | **Antibody** | **Manufacturer** | **Host** | **catalog number** | **Concentração**  **(v/v)** |
| --- | --- | --- | --- | --- | --- |
| Primary | GAD65 | Abcam | Mouse | AB26113 | 1:750 |
|  | GFAP | Abcam | Rabbit | AB7260 | 1:750 |
|  | Iba 1 | Wako | Rabbit | 019-19741 | 1:750 |
|  | Human mitochondria | Abcam | Mouse | Ab92842 | 1:300 |
|  | Synaptophysin | Novus Biological | Rabbit | NBP2-25170 | 1:1000 |
|  | VGluT1 | Synaptic systems | Rabbit | 135303 | 1:1000 |
|  | NeuN | Millipore | Mouse | MAB377 | 1:500 |
|  | Phospho-FGFR | Invitrogen | Rabbit | 105938 | 1:200 |
| Secondary | Alexa Fluor® 488 Anti-Mouse | Jackson ImmunoResearch | Monkey | 715-545-150 | 1:250 |
|  | Alexa Fluor® 488 anti-rabbit | Jackson ImmunoResearch | Monkey | 711-545-152 | 1:250 |
|  | Cy™3 anti-mouse | Jackson ImmunoResearch | Monkey | 715-165-150 | 1:125 |
|  | Cy™3 anti-rabbit | Jackson ImmunoResearch | Donkey | 711-165-152 | 1:250 |

List with manufacturing data, host and concentrations of the antibodies used in this article.

Supplementary Figure 1


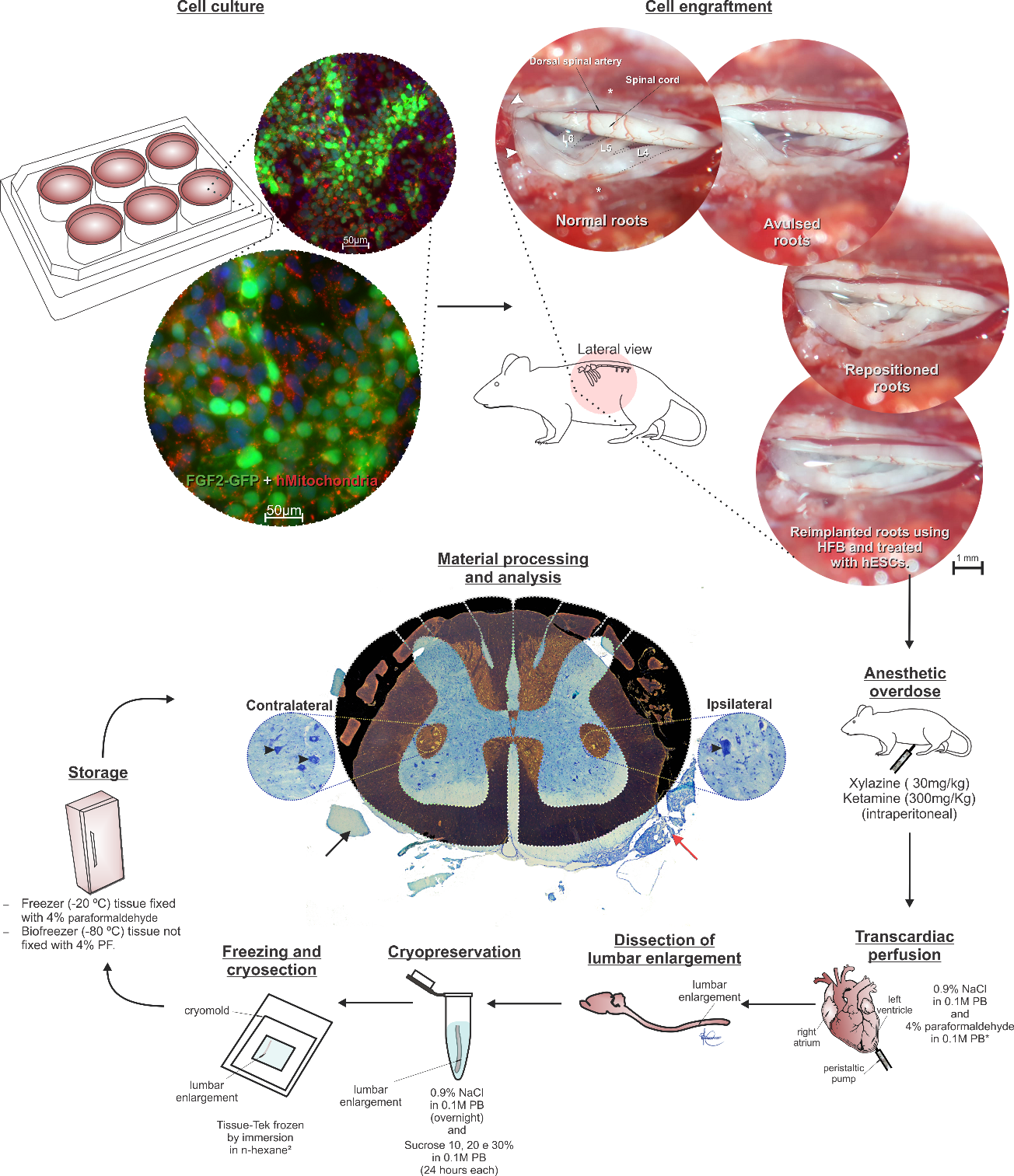


**Supplementary Figure 1.** Workflow used throughout the study. The hESCs (marked in red with anti-human mitochondrial IgG) were cultured and activated with doxycycline to express FGF2 (marked in green) in culture and implanted together with HFB in the region where the avulsion of roots from L4 to L6 occurred. After an adequate survival period, the animals were euthanized, and their tissues were perfused following the appropriate protocol for the technique in question, to allow later analysis, as in the morphology techniques, which involve locating motor neurons residing in laminae IX Rexed on both spinal cord antimeres. Asterisk: lower back musculature; white arrowhead: folded dura mater; black arrowhead: spinal cord motor neurons; black arrow: intact ventral root; red arrow: connective scar tissue formed after reimplantation with HFB.

Supplementary Figure 2


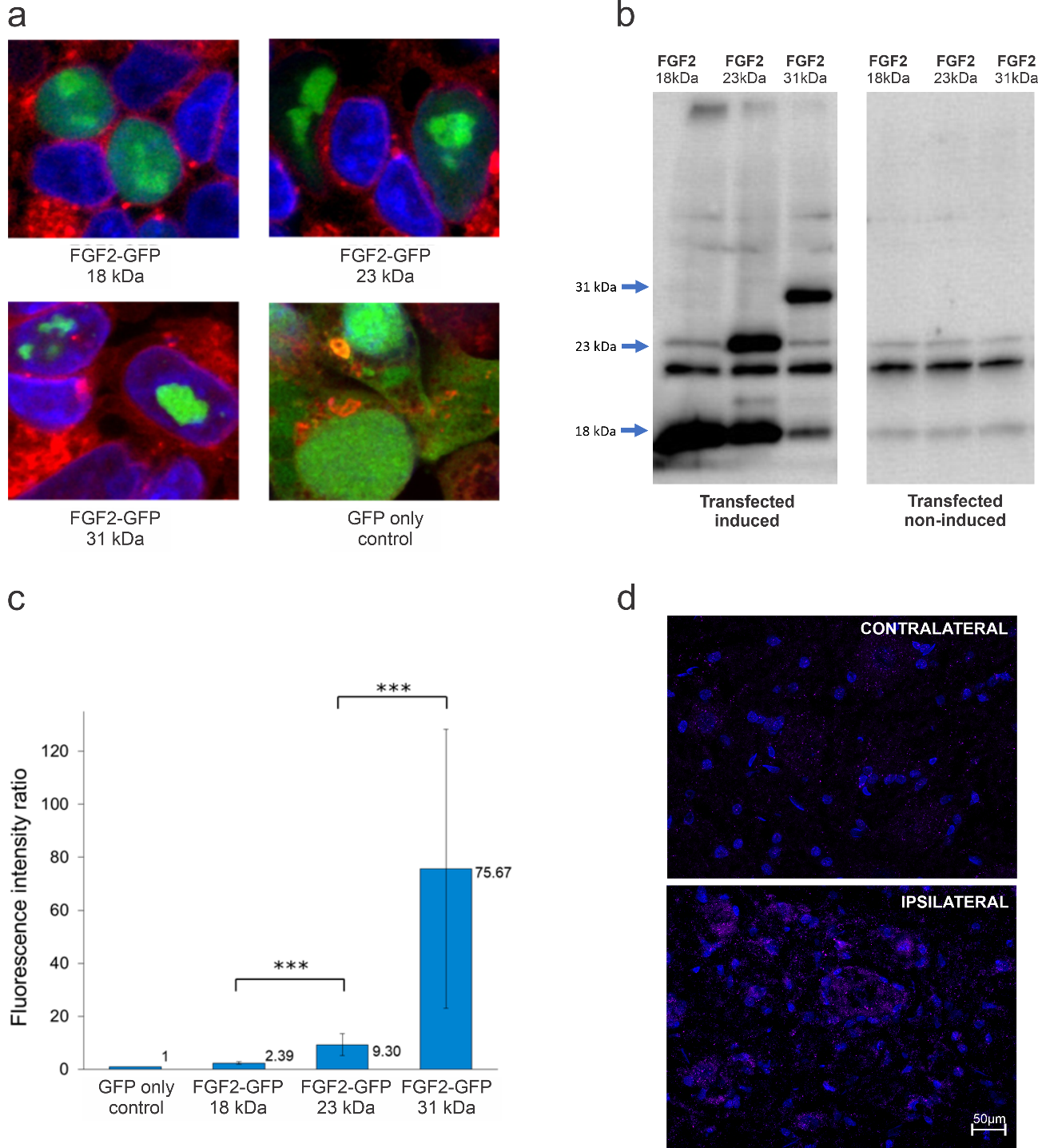


**Supplementary Figure 2.** Intracellular localization of FGF2 isoforms in hESCs. (A), transiently transfected hESC as indicated were labeled with cytoplasmic dye MitoTracker Red, fixed and stained with nuclear DAPI and to be observed under confocal microscopy. Blue (DAPI), green (GFP), and red (mitochondria in the cytoplasm). (B) Western blotting analysis of the FGF2 overexpression (18, 23, 31KDa) in hESCs induced by doxycycline. Observe that 31kDA transfected-induced cells overexpress significantly enhanced amounts of high molecular weight FGF2, although small quantities of the other isoforms are still detectable. (C) The mean intensity of the GFP fluorescence was calculated in nucleoli versus the rest of the nuclei (****p*<0.001). Note the sharp border between green nucleoli and blue-colored nuclei in the case of 31 kD FGF2. (D). Pan-FGF receptor labeling (magenta) following the avulsion of ventral roots. Observe the upregulation ipsilateral to the lesion in comparison to the contralateral side. Blue channel (DAPI).

Supplementary Figure 3


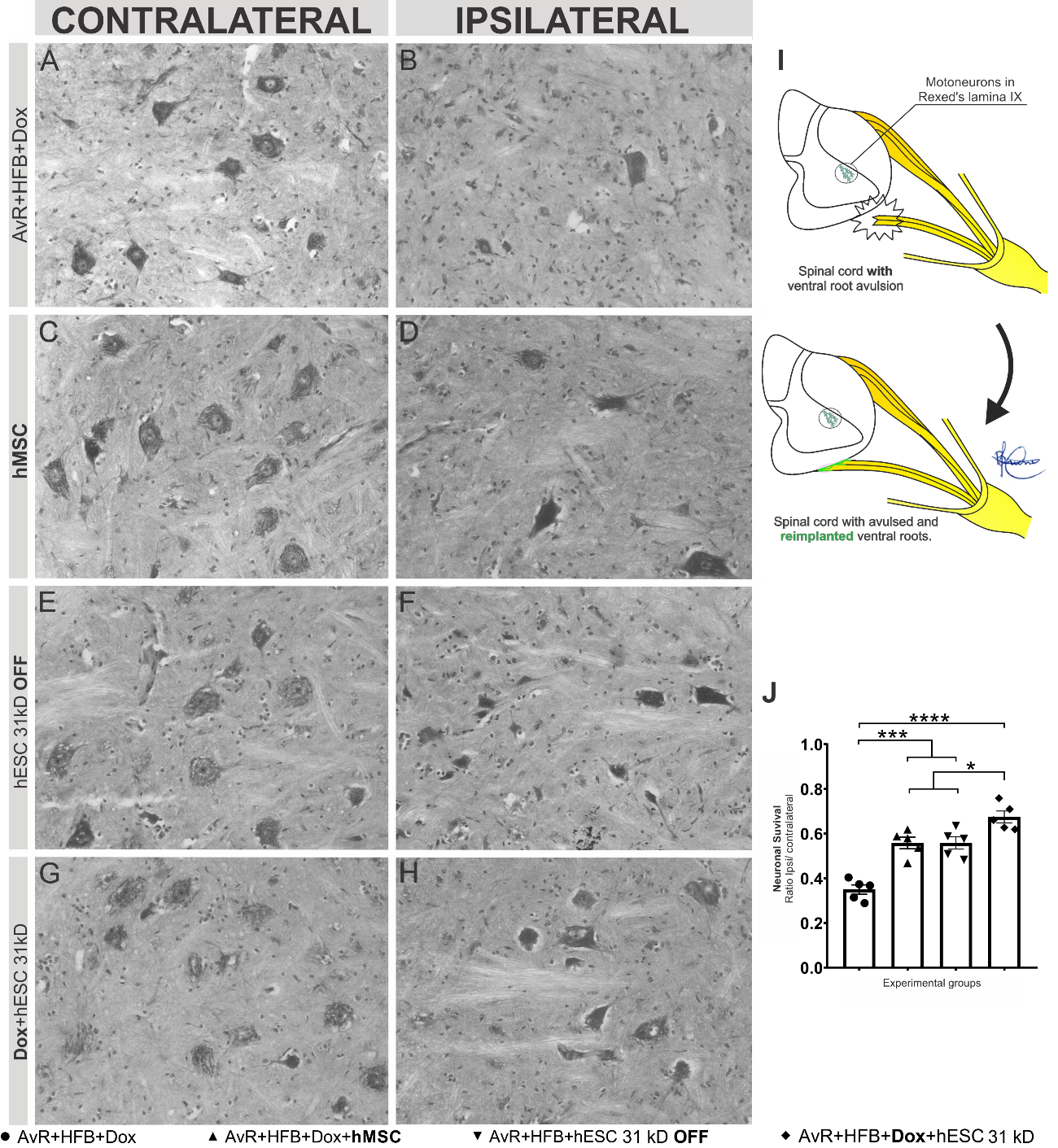


**Supplementary Figure 3.** Assessment of neuronal survival by 31kD FGF2 - hESCs following root avulsion and reimplantation. (A – H) Representative images of the contra and ipsilateral side of all groups. (I) The ventral roots were reimplanted and the animals followed for 12 weeks. (J) Graph quantification of neuronal survival. The group treated with 31kD hESCs demonstrates enhanced preservation of motoneurons after 12 weeks of root repair, indicating long-term preservation of the motor pool.
